# Safety and immunogenicity of 3 doses of BNT162b2 and CoronaVac in children and adults with inborn errors of immunity

**DOI:** 10.3389/fimmu.2022.982155

**Published:** 2022-09-20

**Authors:** Daniel Leung, Xiaofeng Mu, Jaime S. Rosa Duque, Samuel M. S. Cheng, Manni Wang, Wenyue Zhang, Yanmei Zhang, Issan Y. S. Tam, Toby S. S. Lee, Jennifer H. Y. Lam, Sau Man Chan, Cheuk Hei Cheang, Yuet Chung, Howard H. W. Wong, Amos M. T. Lee, Wing Yan Li, Sara Chaothai, Leo C. H. Tsang, Gilbert T. Chua, Kai-Ning Cheong, Elaine Y. L. Au, Janette S. Y. Kwok, Koon Wing Chan, Patrick C. Y. Chong, Pamela P. W. Lee, Marco H. K. Ho, Tsz Leung Lee, Wenwei Tu, Malik Peiris, Yu Lung Lau

**Affiliations:** ^1^ Department of Paediatrics and Adolescent Medicine, The University of Hong Kong, Hong Kong, Hong Kong SAR, China; ^2^ School of Public Health, The University of Hong Kong, Hong Kong, Hong Kong SAR, China; ^3^ Hong Kong Children’s Hospital, Hong Kong, Hong Kong SAR, China; ^4^ Division of Clinical Immunology, Department of Pathology, Queen Mary Hospital, Hong Kong, Hong Kong SAR, China; ^5^ Division of Transplantation and Immunogenetics, Department of Pathology, Queen Mary Hospital, Hong Kong, Hong Kong SAR, China; ^6^ Virtus Medical, Hong Kong, Hong Kong SAR, China; ^7^ Centre for Immunology and Infection C2i, Hong Kong, Hong Kong SAR, China

**Keywords:** BNT162b2, CoronaVac, COVID-19, inborn errors of immunity, vaccine

## Abstract

Our study (NCT04800133) aimed to determine the safety and immunogenicity in patients with IEIs receiving a 3-dose primary series of mRNA vaccine BNT162b2 (age 12+) or inactivated whole-virion vaccine CoronaVac (age 3+) in Hong Kong, including Omicron BA.1 neutralization, in a nonrandomized manner. Intradermal vaccination was also studied. Thirty-nine patients were vaccinated, including 16 with homologous intramuscular 0.3ml BNT162b2 and 17 with homologous intramuscular 0.5ml CoronaVac. Two patients received 3 doses of intradermal 0.5ml CoronaVac, and 4 patients received 2 doses of intramuscular BNT162b2 and the third dose with intradermal BNT162b2. No safety concerns were identified. Inadequate S-RBD IgG and surrogate virus neutralization responses were found after 2 doses in patients with humoral immunodeficiencies and especially so against BA.1. Dose 3 of either vaccine increased S-RBD IgG response. T cell responses against SARS-CoV-2 antigens were detected in vaccinated IEI patients by intracellular cytokine staining on flow cytometry. Intradermal third dose vaccine led to high antibody response in 4 patients. The primary vaccination series of BNT162b2 and CoronaVac in adults and children with IEIs should include 3 doses for optimal immunogenicity.

## Graphical Abstract

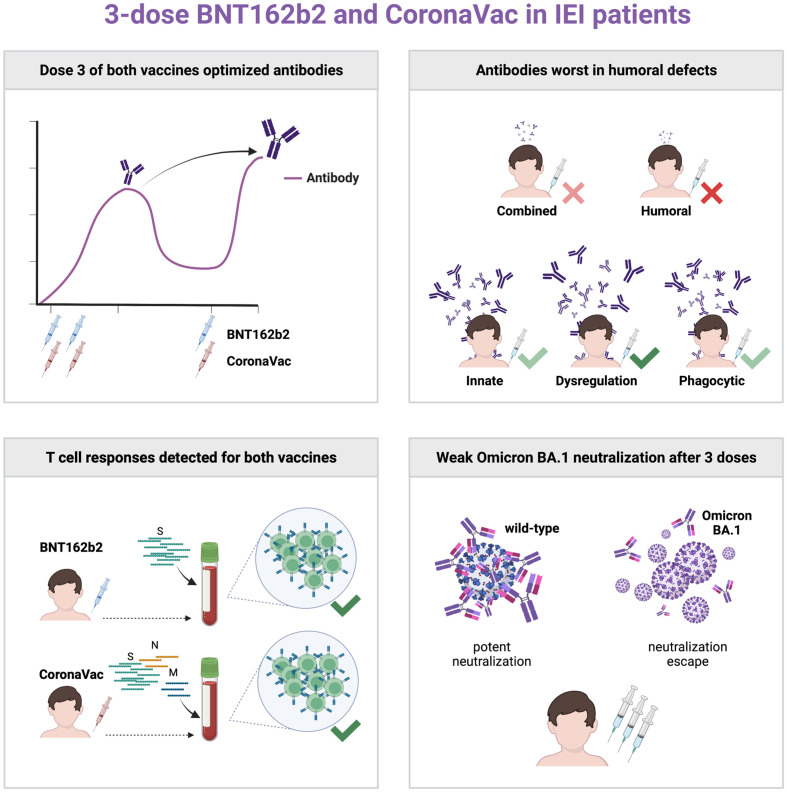

## Introduction

The COVID-19 pandemic has disproportionately affected patients with inborn errors of immunity (IEIs) and other forms of immune compromise and dysregulation, with higher mortality (1–4), rare presentations such as multisystem inflammatory syndrome in children (MIS-C) (5, 6), and delayed viral clearance reported (7, 8). Inborn errors of type I interferon pathway and autoantibody phenocopies have also been found to cause a substantial proportion of previously healthy patients with life-threatening COVID-19 pneumonia (9–11).

mRNA and inactivated COVID-19 vaccines protect strongly against severe outcomes of COVID-19 at the population level (12, 13). Patients with IEIs or immune dysregulation likely have variable levels of vaccine efficacy, especially for those with defects in adaptive immunity. The initial licensing COVID-19 vaccine trials excluded patients with immunocompromise, driving the need for post-licensure studies to study the immunogenicity of COVID-19 vaccines in IEI patients.

Several studies have reported the immunogenicity of COVID-19 vaccines in IEI patients, but most only focused on 2 doses of COVID-19 mRNA vaccines in adult patients with IEI, mainly common variable immunodeficiency (CVID) (14–19). Inactivated vaccines against COVID-19 such as CoronaVac are extensively used worldwide with more than 4 billion doses distributed, and differ in their ability to induce antibody and T cell responses compared to mRNA vaccines (20). Moreover, children and adolescents respond to SARS-CoV-2 infection and vaccination differently than adults (21, 22), and we previously reported that adolescents elicit higher antibody response to BNT162b2 and CoronaVac compared with adults (20). Currently in Hong Kong, immunocompromised patients above the age of 5 are recommended to receive a 3-dose mRNA or inactivated vaccine primary series; or, for those aged 3-4, a 3-dose inactivated vaccine primary series, based on poorer immunogenicity and clinical vulnerability (23). However, the immunogenicity of three doses of vaccine in patients with heterogeneous IEI remain unclear. In addition, intradermal vaccination of some vaccines such as seasonal flu vaccine has been trialed in immunocompromised patients or older adults, to enhance immunogenicity (24). This represents a potential option for enhanced immunogenicity of COVID-19 vaccination in patients of IEI as well.

The Omicron BA lineage poses a public health threat with enhanced transmissibility and escape from virus neutralization (25, 26), reducing the effectiveness of COVID-19 vaccines against symptomatic disease but less so for severe outcomes (12). While T cell epitopes are believed to be preserved (27), Omicron neutralization in vaccinated IEI patients is unknown.

To address these questions, we initiated a 3-year nonrandomized study (NCT04800133) to study the safety and immunogenicity of COVID-19 vaccines in children and adults receiving mRNA COVID-19 vaccine BNT162b2 or inactivated whole-virion vaccine CoronaVac in Hong Kong. In the present interim analysis, we focus on patients with IEI who received 3-doses of BNT162b2 (aged 12 and above) or CoronaVac (aged 3 and above). Adverse reactions (ARs) and adverse events (AEs) were monitored after each dose, and humoral and cellular immunogenicity against the wild-type (WT) SARS-CoV-2, as well as neutralization capacity against Omicron BA.1 in IEI patients were studied. Cases of Omicron BA.2 breakthrough infections were also described.

## Methods

### Study design

COVID-19 Vaccination in Adolescents and Children (COVAC; NCT04800133) is a non-randomized study aimed at investigating the immunogenicity of BNT162b2 and CoronaVac, in healthy children and immunocompromised patients as previously described (20, 28, 29). The study was approved by the University of Hong Kong (HKU)/Hong Kong West Cluster Hospital Authority Institutional Review Board (UW21-157).

### Participants

This prespecified interim analysis included patients aged 3 years and above who were diagnosed with IEIs and received at least one dose of COVID-19 vaccine. Participants with no known history of IEIs were excluded from this analysis.

### Procedures

Potential participants were IEI patients diagnosed by the Department of Pediatrics and Adolescent Medicine, Queen Mary Hospital, The University of Hong Kong. YLL and JSRD obtained informed consent from participants aged 18 years or above. Underage participants provided informed assent, and consent was obtained from their parents or legally acceptable representatives. Patients were free to elect a homologous intramuscular primary series, a homologous intradermal primary series, or a heterologous primary series with 2 doses of intramuscular vaccine followed by intradermal dose 3. Three doses of 0.3ml BNT162b2 or 0.5 ml CoronaVac were administered *via* the intramuscular route to the deltoid or anterolateral thigh, or by an intradermal inoculator (MicronJet600, NanoPass Technologies, Nes Ziona, Israel) to the deltoid. Doses 2 and 3 were given at least 14 and 28 days after the preceding dose.

#### Safety data collection

Participants were observed by a study nurse or pediatrician for at least 15 minutes after vaccination. Participants reported prespecified adverse reactions (ARs) in an online or paper-based diary for 7 days after vaccination. Anti-pyretic use was also solicited for 7 days after vaccination, and participants were recommended not to use anti-pyretics to prevent the onset of ARs. Unsolicited adverse events (AEs) were captured for up to 28 days after vaccination. Severe AEs, involving hospitalizations, life-threatening complications, disabilities, deaths and birth defects of their offspring, or breakthrough COVID-19, would be monitored for 3 years after vaccination. Adverse events reported were reviewed by investigators, who determined any possibility of causal relationship with the study vaccine.

#### Immunogenicity

Immunogenicity outcomes were evaluated at baseline (pre-dose 1), post-dose 1 (pre-dose 2, 21-28 days after dose 1), post-dose 2 (28 days after dose 2), pre-dose 3 (at least 28 days after dose 2), and post-dose 3 (28 days after dose 3). Primary humoral immunogenicity outcomes include wild-type (WT) Spike receptor-binding domain (S-RBD) IgG ELISA and WT surrogate virus neutralization test (sVNT), and secondary outcomes include BA.1 sVNT. WT S-RBD IgG enzyme-linked immunosorbent assay (ELISA) were carried out as previously described and validated (20, 30). sVNT was conducted according to the manufacturer’s instructions (GenScript Inc, Piscataway, USA) and as described in our previous publications (30). IFN-γ^+^ or IL-2^+^ CD4^+^ or CD8^+^ T cell responses were examined by intracellular cytokine staining after stimulation with SARS-CoV-2 S peptide pool (and N and M peptide pools for CoronaVac recipients) (Miltenyi Biotec, Bergisch Gladbach, Germany) as a primary cellular outcome as described previously (20, 29). Frequencies of T cell responses against S, N and M peptide pools are added together for CoronaVac recipients. Negative values, i.e., below the limit of detection (LOD), limit of quantification (LOQ) or cut-off, are imputed as half the limit/cut-off. All available results from IEI patients who received at least one dose, prior to the analysis, were presented. Results from timepoints after breakthrough infection were excluded from disease group and longitudinal analyses. Additional details are available in the Supplemental Methods.

## Results

### Participant composition

A total of 39 patients, aged between 5-51 years (median 17 years), received at least one dose of COVID-19 vaccine, including 16 with homologous intramuscular BNT162b2 vaccine (age range 13-50 years, median 19 years); 17 with homologous intramuscular CoronaVac vaccine (age range 5-51 years, median 15 years). Two elected to receive 3 doses of intradermal CoronaVac, and 4 received 2 doses of intramuscular BNT162b2 and a third dose being intradermal BNT162b2 injection. Patients had a range of IEIs, and their demographics and clinical characteristics are summarized in **Table 1**. That included 5 patients with combined defects (3 received BNT162b2, 2 received CoronaVac), 5 patients with innate defects (4 BNT162b2, 1 CoronaVac), 17 patients with humoral defects (6 BNT162b2, 11 CoronaVac), 5 patients with dysregulation (3 BNT162b2, 2 CoronaVac), and 7 patients with phagocytic defects (4 BNT162b2, 3 CoronaVac). None reported SARS-CoV-2 infection prior to immunization and all available baseline sera tested negative for S-RBD IgG.

**Table 1:** Participant profile.

Patient No.	Vaccine brand	Route	Disease category	Age	Sex	Diagnosis	Mutation	COVID-19 breakthrough	Current medications	ALC10^9^/L	CD3/ul	CD19/ul	IgGmg/dl
1	BB	MM	Dysregulation	19	M	HIDS	AR *MVK*:c.928G>A,p.V310M	1 mo after dose 3	Anakinra, MTX	3.14	/	/	NA
2	BB	MM	Humoral	29	M	XLA	XL *BTK*:c.1567-2A>T (splicing)	3 mo before dose 3	SCIG	1.55	2282	0	1075
3	BB	MM	Humoral	28	M	XLA	XL *BTK*:c.942A>G (splicing)	/	SCIG	1.25	1897	1	1241
4	BB	MM	Innate	28	M	STAT1 GOF	AD *STAT1*:c.800C>T,p.A267V	/	/	NA	1570	121	NA
5	BB	MM	Phagocytic	16	M	X-CGD, post-HSCT	XL *CYBB*:c.1437C>A,p.Y479*	/	/	NA	5022	2427	NA
6	BBB	MMD	Combined	16	F	DN STAT3	AD *STAT3*:c.2134T>C,p.C712R	/	IVIG	1.99	3928	1435	NA
7	BBB	MMD	Dysregulation	26	M	XMEN	XL *MAGT1*:c.916delC,p.L306fs	/	IVIG	1.43	802	400	939
8	BBB	MMD	Humoral	13	M	XLA	XL *BTK*:c.1696C>T,p.P566S	/	IVIG	5.55	3310	15	1020
9	BBB	MMD	Innate	15	M	STAT1 GOF	AD *STAT1*:c.1170G>A,p.M390I	/	/	NA	1328	352	NA
10	BBB	MMM	Combined	21	M	AT	/	/	IVIG	0.92	508	6	824
11	BBB	MMM	Combined	49	M	XLT	XL *WAS*:c.134C>T,p.T45M	/	/	1.2	/	/	NA
12	BBB	MMM	Dysregulation	18	F	HLH	/	/	IVIG, MMF, pred	0.52	364	115	803
13	BBB	MMM	Humoral	34	M	XLA	XL *BTK*:c.887del,p.K296Sfs*35	1 wk after dose 3	IVIG	1.01	780	0	1089
14	BBB	MMM	Humoral	13	M	XLA	XL *BTK*:c.1111T>C,p.S371P	/	IVIG	1.8	2244	2	750
15	BBB	MMM	Humoral	30	M	XLA	XL *BTK*:c.41C>A,p.S14Y	/	IVIG	1.7	/	/	1044
16	BBB	MMM	Innate	30	M	STAT1 GOF	AD *STAT1*:c.1151G>A,p.G384D	/	/	0.99	883	99	1148
17	BBB	MMM	Innate	19	F	STAT1 GOF	AD *STAT1*:c.1074G>T,p.L358F	/	/	0.45	358	0	1111
18	BBB	MMM	Phagocytic	50	F	X-CGD carrier	XL *CYBB*:c.469C>T,p.R157*	/	/	1.59	/	/	NA
19	BBB	MMM	Phagocytic	23	M	X-CGD, post-HSCT	XL *CYBB*:c.1498G>C,p.D500H	/	/	2.66	2184	256	1077
20	BBB	MMM	Phagocytic	14	M	X-CGD	XL *CYBB*:c.469C>T,p.R157*	/	/	2.17	1506	885	NA
21	CC	MM	Combined	13	M	XLT	XL *WAS*:c.116T>G,p.L39R	/	/	NA	2273	675	NA
22	CC	MM	Humoral	5	F	Agamma	/	2 wk after dose 1	IVIG	1.32	1129	11	1119
23	CC	MM	Humoral	11	M	XLA	XL *BTK*:c.3G>T,p.M1I	/	IVIG	2.58	8594	9	814
24	CC	MM	Humoral	30	M	XLA	XL *BTK*:c.3G>T,p.M1I	/	IVIG	2.14	/	/	1409
25	CC	MM	Phagocytic	10	F	AR-CGD, post-HSCT	AR *CYBA*:c.371C>T,p.A124V	/	/	2.05	1353	395	973
26	CCC	DDD	Combined	15	M	X-SCID, post-HSCT	XL *IL2RG*:c.562C>T,p.Q188*	1 mo before dose 3	/	2.23	1172	300	1289
27	CCC	DDD	Innate	8	M	CARD9	AR het *CARD9*:c.586A>G,p.K196E and c.1526G>A,p.R509K	/	/	1.91	1915	347	NA
28	CCC	MMM	Dysregulation	8	M	CINCA	AD *NLRP3*:c.1711G>C,p.G571R	/	Canakinumab	NA	/	/	NA
29	CCC	MMM	Dysregulation	15	M	CINCA	AD somatic mosaicism *NLRP3*:c. 998G>T,p.S333I	1 mo after dose 3	MTX, canakinumab	1.6	1096	303	1361
30	CCC	MMM	Humoral	5	M	XLA	XL *BTK*:c.1559G>C,p.R520P	/	IVIG	2.7	5465	10	717
31	CCC	MMM	Humoral	7	M	XLA	XL *BTK*:c.1559G>C,p.R520P	/	IVIG	2	2212	15	613
32	CCC	MMM	Humoral	19	F	CVID	/	/	IVIG, HCQ, MMF, pred	2.14	1178	184	864
33	CCC	MMM	Humoral	18	M	XLA	XL *BTK*:c.332T>C,p.L111P	/	IVIG	1.7	3552	6	NA
34	CCC	MMM	Humoral	32	M	XLA	XL *BTK*:c.41C>A,p.S14Y	/	IVIG	1.8	1698	4	1076
35	CCC	MMM	Humoral	14	M	XLA	XL *BTK*:c.947_948del,p.T316Sfs*6	/	IVIG	3.04	4456	1	1279
36	CCC	MMM	Humoral	15	M	XLA	XL *BTK*:c.1535T>C,p.L512P	3 mo after dose 3	IVIG	2.12	2399	8	916
37	CCC	MMM	Humoral	34	M	XLA	XL *BTK :* EX2-EX3del	/	IVIG	2.7	/	/	NA
38	CCC	MMM	Phagocytic	11	M	SCN	AD *ELA2*:c.362T>C,p.L121P	/	G-CSF	3.12	/	/	NA
39	CCC	MMM	Phagocytic	51	M	SCN	AD *ELA2*:c.362T>C,p.L121P	/	/	NA	/	/	NA

Current immunomodulatory medications, absolute lymphocyte count (ALC) and IgG level around the time of dose 1 are provided. CD3 and CD19 counts are the last available measurements before vaccination. B, BNT162b2; C, CoronaVac; M (route), intramuscular; D, intradermal; M (sex), male; F, female; HIDS, HyperIgD syndrome; XLA, X-linked agammaglobulinemia; X-CGD, X-linked chronic granulomatous disease; HSCT, hemopoietic stem cell transplant; DN-STAT3, dominant-negative STAT3 disease; X-MEN, X-linked immunodeficiency with magnesium defect; Epstein-Barr virus infection, and neoplasia; AT, ataxia telangiectasia; XLT, X-linked thrombocytopenia; HLH, hemophagocytic lymphohistiocytosis; AR-CGD, autosomal recessive chronic granulomatous disease; CINCA - chronic infantile, neurologic, cutaneous, and articular syndrome; CVID, common variable immunodeficiency; SCN, severe congenital neutropenia; MTX, methotrexate; SCIG, subcutaneous immunoglobulin; IVIG, intravenous immunoglobulin; MMF, mycophenolate mofetil; pred, prednisolone; HCQ, hydroxychloroquine; G-CSF, granulocyte-colony stimulating factor; NA, not available.

### Safety outcomes

**Figure 1:**
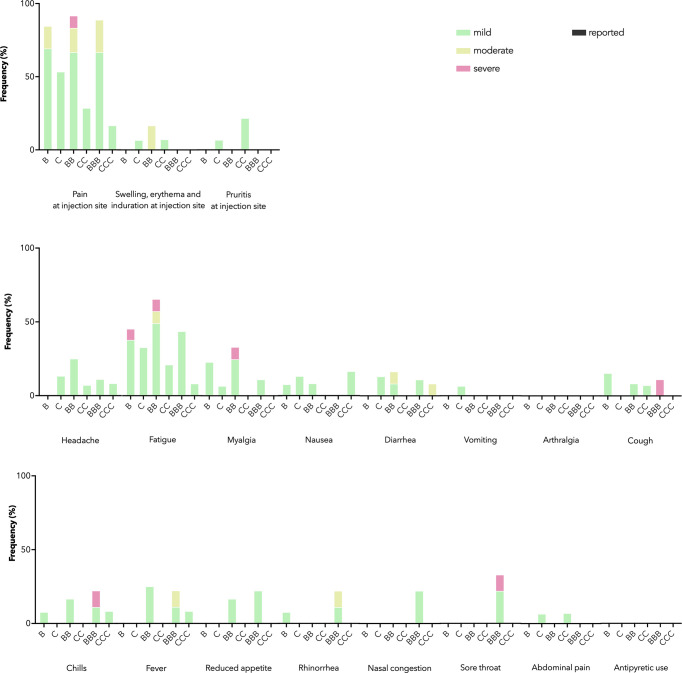
Adverse reactions (ARs) and antipyretic use reported 7 days after each dose by vaccine brand. B, BB, and BBB refer to 1, 2, and 3 doses of BNT162b2 while C, CC, and CCC refer to 1, 2, and 3 doses of CoronaVac. Stacked bar chart shows ARs by maximal severity in different colors. Severity was self-graded by participants, according to whether the AR affected daily activity (mild – tolerable, not affecting daily activities, moderate - performance of some daily activities affected, or severe - performance of some daily activities prevented), or for swelling, erythema and induration at injection site, the diameter affected (mild – 2-5 cm, moderate – 5-10 cm, severe – above 10 cm), or for fever, the body temperature (mild – 38.0-38.4°C, moderate – 38.5-38.9°C, severe – 39.0 °C or above).

As a primary safety outcome, we tracked ARs for 7 days and AEs for 28 days after each dose. IEI patients receiving both vaccines mainly reported mild and moderate ARs (**Figure 1**). Severe ARs, e.g. pain at injection site and fatigue, were reported in some patients receiving BNT162b2 but not CoronaVac, in line with previous findings in healthy adolescents of milder reactogenicity with CoronaVac (20). Eight AEs were reported within 28 days after BNT162b2 (n=4) or CoronaVac (n=4), including rash (n=3), chest discomfort (n=3), nodule in tongue (n=1) and lymphadenopathy (n=1), which were all of mild severity. One patient reported two non-fatal resolved severe AE (asthma exacerbation and poisoning respectively) 107 days after dose 2 and 73 days after dose 3 of BNT162b2. Both severe AEs were deemed not relevant to study vaccination.

### S-RBD IgG and surrogate virus neutralization by disease category

**Figure 2:**
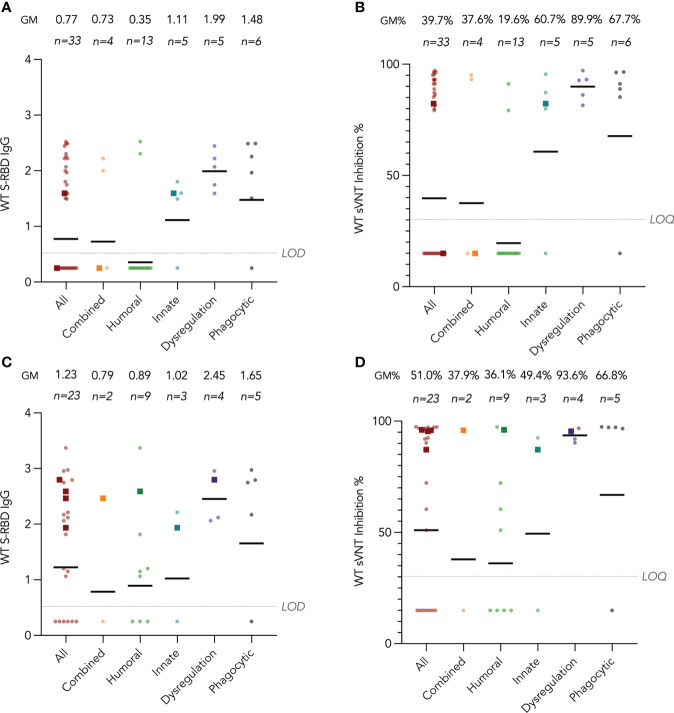
Wild-type (WT) Spike-receptor binding domain (S-RBD) IgG and surrogate virus neutralization test (sVNT) results after COVID-19 vaccination by disease category. **(A)** and **(B)** show S-RBD IgG and sVNT results respectively in five disease categories (combined, humoral, innate, dysregulation and phagocytic) after 2 doses, while C and D show S-RBD and sVNT results after 3 doses. Geometric means (GM) are shown with centre lines and stated above each column. Limits of detection and quantification (LOD and LOQ) were drawn as grey lines, and the y-axis shows the range of the assays. Number of analyzed available samples (n) are also stated above each column. A and B included 2 patients (one each in combined and innate) who received first 2 doses intradermally and their datapoints were shown as darkened squares. **(C)** and **(D)** also included 4 patients (1 each in combined, humoral, innate and dysregulation) who received their third dose intradermally and their datapoints were also shown as darkened squares. .

Humoral immunogenicity outcomes including WT S-RBD IgG and WT sVNT were analyzed at post-dose 2 and post-dose 3 timepoints by disease category in **Figure 2A–D** and listed in **Table 2**. Overall, 55% (18 out of 33 with available results) and 74% (17 out of 23) patients were seropositive (i.e., detectable S-RBD IgG) at post-dose 2 and post-dose 3 respectively. As expected, patients with humoral deficiency had the lowest geometric mean (GM) S-RBD IgG level (**Figure 2A**) and sVNT inhibition (**Figure 2B**) with 15% (2 of 13) seropositive at the post-dose 2 timepoint. A total of 15 patients, who had X-linked severe combined immunodeficiency (X-SCID) post-hematopoietic stem cell transplant (HSCT) (n=1), *STAT1* gain-of-function (n=1), severe congenital neutropenia (SCN, n=1), ataxia telangiectasia (n=1), and humoral immunodeficiencies (n=11), did not respond and were seronegative after 2 doses, suggesting these IEIs were associated with seroconversion failure (**Figure 2A**). Notably, among those with a humoral deficiency, two brothers with X-linked agammaglobulinemia (XLA) seroconverted after dose 2. Six more patients with CVID (n=1) and XLA (n=5) also seroconverted after dose 3 (**Figure 2C**), despite the modest level of neutralization attained (**Figure 2D**). The highest neutralization responses were observed in patients with immune dysregulation with GM WT sVNT inhibition of 93.6% post-dose 3 (**Figure 2D**). Longitudinal analyses of WT S-RBD IgG and WT sVNT could be found in **Supplementary Materials** (**Figure S1A, B**).

**Table 2:** Antibody responses in IEI patients.

Patient No.	Vaccine brand	Route	Disease category	S-RBD IgG post-dose 2	WT sVNT post-dose 2 (%)	BA.1 sVNT post-dose 2 (%)	S-RBD IgG post-dose 3	WT sVNT post-dose 3 (%)	BA.1 sVNT post-dose 3 (%)
1	BB	MM	Dysregulation	2.07	97.14	38.09	/	/	/
2	BB	MM	Humoral	0.25	15.00	10.00	/	/	/
3	BB	MM	Humoral	0.25	15.00	10.00	/	/	/
4	BB	MM	Innate	1.81	95.50	10.00	/	/	/
6	BBB	MMD	Combined	2.00	93.15	10.00	2.47	95.87	10.00
7	BBB	MMD	Dysregulation	2.22	92.72	10.00	2.80	95.43	10.00
8	BBB	MMD	Humoral	/	/	/	2.59	96.09	31.22
9	BBB	MMD	Innate	1.60	87.19	10.00	1.93	87.18	/
10	BBB	MMM	Combined	0.25	15.00	10.00	0.25	15.00	10.00
11	BBB	MMM	Combined	2.22	95.06	10.00	/	/	/
12	BBB	MMM	Dysregulation	1.59	81.51	10.00	2.96	96.79	/
13	BBB	MMM	Humoral	0.25	15.00	10.00	0.25*	15.00*	/
14	BBB	MMM	Humoral	0.25	15.00	10.00	1.82	72.21	/
15	BBB	MMM	Humoral	0.25	15.00	10.00	3.37	97.32	68.88
16	BBB	MMM	Innate	1.49	79.97	10.00	2.21	92.45	/
17	BBB	MMM	Innate	0.25	15.00	10.00	0.25	15.00	10.00
18	BBB	MMM	Phagocytic	2.49	91.22	10.00	2.79	97.21	10.00
19	BBB	MMM	Phagocytic	1.97	96.51	21.88	2.97	96.61	88.19
20	BBB	MMM	Phagocytic	2.49	96.25	10.00	2.75	97.23	85.47
22	CC	MM	Humoral	0.25*	15.00*	/	/	/	/
23	CC	MM	Humoral	0.25	15.00	/	/	/	/
25	CC	MM	Phagocytic	2.26	85.24	/	/	/	/
26	CCC	DDD	Combined	0.25	15.00	10.00	0.25*	15.00*	/
27	CCC	DDD	Innate	1.59	82.25	/	/	/	/
28	CCC	MMM	Dysregulation	2.44	86.13	/	2.12	90.25	/
29	CCC	MMM	Dysregulation	1.75	93.07	10.00	2.06	92.00	28.96
30	CCC	MMM	Humoral	2.30	79.20	/	/	/	/
31	CCC	MMM	Humoral	2.52	91.19	/	/	/	/
32	CCC	MMM	Humoral	/	/	/	1.15	60.42	/
33	CCC	MMM	Humoral	0.25	15.00	10.00	0.25	15.00	10.00
34	CCC	MMM	Humoral	0.25	15.00	10.00	1.20	50.96	10.00
35	CCC	MMM	Humoral	0.25	15.00	10.00	0.25	15.00	10.00
36	CCC	MMM	Humoral	0.25	15.00	10.00	1.06	15.00	10.00
37	CCC	MMM	Humoral	0.25	15.00	10.00	0.25	15.00	10.00
38	CCC	MMM	Phagocytic	1.51	88.88	10.00	2.17	97.42	/
39	CCC	MMM	Phagocytic	0.25	15.00	10.00	0.25	15.00	/

* denotes results samples taken after breakthrough infection. S-RBD IgG and sVNT results are given as OD450 values and % inhibition respectively. Negative S-RBD IgG, WT sVNT and BA.1 sVNT results were imputed as 0.25, 15 and 10 respectively. B, BNT162b2; C, CoronaVac; M (route), intramuscular; D, intradermal.

### T cell responses by disease category

**Figure 3:**
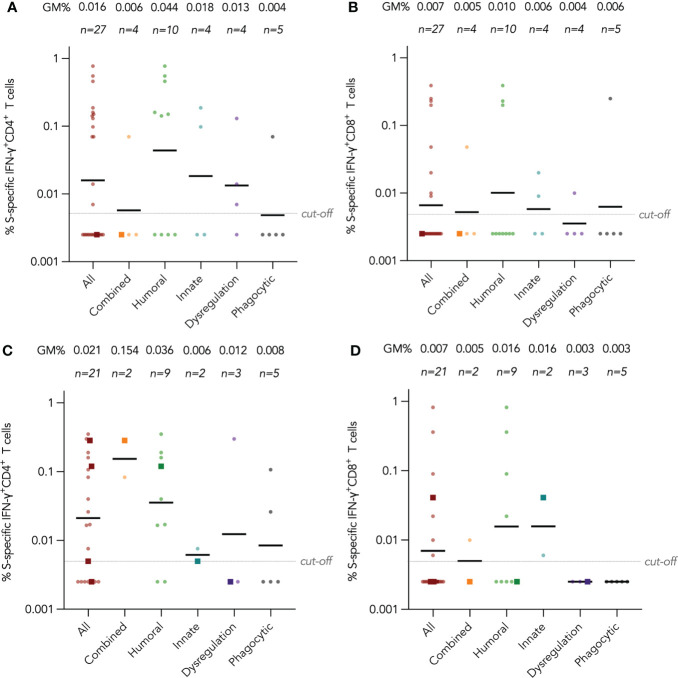
Wild-type (WT) S peptide pool-specific interferon-γ (IFN-γ)^+^ CD4^+^ and CD8^+^ T cells after COVID-19 vaccination by disease category. **(A)** and **(B)** show S-specific IFN-γ^+^ CD4^+^ and CD8^+^ results respectively in five disease categories (combined, humoral, innate, dysregulation and phagocytic) after 2 doses, while C and D show S-specific IFN-γ^+^ CD4^+^ and CD8^+^ results after 3 doses. Geometric means (GM) are shown with center lines and stated above each column. Cut-offs were drawn as grey lines. Number of analyzed available samples (n) are also stated above each column. A and B included 1 patient (in combined) who received first 2 doses intradermally and their datapoints were shown as darkened squares. **(C)** and **(D)** also included 4 patients (1 each in combined, humoral, innate and dysregulation) who received their third dose intradermally and their datapoints were also shown as darkened squares.

IFN-γ^+^ and IL-2^+^ CD4^+^ and CD8^+^ T cell responses were examined by intracellular cytokine staining after stimulation with SARS-CoV-2 S peptide pool (and N and M peptide pools for CoronaVac recipients) as primary cellular outcomes, as tabulated in **Table 3**. We analyzed S-specific IFN-γ^+^ CD4^+^ and CD8^+^ T cell responses at post-dose 2 and post-dose 3 timepoints by disease category in **Figures 3A–D**; results for S-specific IL-2^+^ CD4^+^ and CD8^+^ T cell responses are shown in the Fig S2A-D. When all IEI patients were considered, 48% and 30% patients respectively had a positive S-specific IFN-γ^+^ CD4^+^ and CD8^+^ T cell after 2 doses, with low geometric mean frequencies of 0.016% and 0.007% (**Figures 3A, B**). As T cell responses after vaccine were also variable in the healthy population, we were not powered to interpret T cell findings in disease categories with very small sample sizes. Patients with humoral deficiencies mounted a robust T cell response with a geometric mean frequency of 0.044% and 0.01% S-specific IFN-γ^+^ CD4^+^ and CD8^+^ T cell responses at the post-dose 2 timepoint. At the post-dose 3 timepoint, 62% and 33% of all patients showed a positive S-specific IFN-γ^+^ CD4^+^ and CD8^+^ T cell responses respectively. Patients with humoral deficiencies had S-specific IFN-γ^+^ CD4^+^ and CD8^+^ T cell responses of 0.036% and 0.016%, respectively, at the post-dose 3 timepoint. Longitudinal analyses of WT S-RBD IgG and WT sVNT could be found in **Supplementary Materials** (**Figure S3A, B**).

**Table 3:** S-specific and SNM-specific IFN-γ^+^ CD4 and CD8 T cell responses in IEI patients.

Patient No.	Vaccine brand	Route	Disease category	S IFN-γ^+^ CD4 post-dose 2 (%)	S IFN-γ^+^ CD8 post-dose 2 (%)	SNM IFN-γ^+^ CD4 post-dose 2 (%)	SNM IFN-γ^+^ CD8 post-dose 2 (%)	S IFN-γ^+^ CD4 post-dose 3 (%)	S IFN-γ^+^ CD8 post-dose 3 (%)	SNM IFN-γ^+^ CD4 post-dose 3 (%)	SNM IFN-γ^+^ CD8 post-dose 3 (%)
1	BB	MM	Dysregulation	0.014	0.0025	/	/	/	/	/	/
2	BB	MM	Humoral	0.46	0.20	/	/	/	/	/	/
3	BB	MM	Humoral	0.14	0.0025	/	/	/	/	/	/
4	BB	MM	Innate	0.19	0.0025	/	/	/	/	/	/
6	BBB	MMD	Combined	0.070	0.0025	/	/	0.28	0.0025	/	/
7	BBB	MMD	Dysregulation	0.0070	0.010	/	/	0.0025	0.0025	/	/
8	BBB	MMD	Humoral	/	/	/	/	0.12	0.0025	/	/
9	BBB	MMD	Innate	0.0025	0.0090	/	/	0.0050	0.041	/	/
10	BBB	MMM	Combined	0.0025	0.0025	/	/	0.083	0.010	/	/
11	BBB	MMM	Combined	0.0025	0.048	/	/	/	/	/	/
12	BBB	MMM	Dysregulation	0.13	0.0025	/	/	0.299	0.0025	/	/
13	BBB	MMM	Humoral	0.55	0.39	/	/	0.19*	0.71*	/	/
14	BBB	MMM	Humoral	0.16	0.0025	/	/	0.35	0.82	/	/
15	BBB	MMM	Humoral	0.0025	0.0025	/	/	0.19	0.0025	/	/
16	BBB	MMM	Innate	0.0025	0.020	/	/	/	/	/	/
17	BBB	MMM	Innate	0.098	0.0025	/	/	0.0076	0.0060	/	/
18	BBB	MMM	Phagocytic	0.0025	0.0025	/	/	0.0025	0.0025	/	/
19	BBB	MMM	Phagocytic	0.0025	0.0025	/	/	0.0025	0.0025	/	/
20	BBB	MMM	Phagocytic	0.0025	0.0025	/	/	0.11	0.0025	/	/
26	CCC	DDD	Combined	0.0025	0.0025	0.16	0.0075	0.32*	0.34*	1.0*	1.4*
29	CCC	MMM	Dysregulation	0.0025	0.0025	0.0075	0.0075	0.0025	0.0025	0.055	0.18
32	CCC	MMM	Humoral	/	/	/	/	0.017	0.090	/	/
33	CCC	MMM	Humoral	0.15	0.0025	0.16	0.0075	0.0025	0.0025	0.0075	/
34	CCC	MMM	Humoral	0.0025	0.0025	0.0075	0.0075	0.16	0.36	0.37	1.0
35	CCC	MMM	Humoral	0.0025	0.0025	0.41	0.53	0.017	0.022	0.053	0.065
36	CCC	MMM	Humoral	0.77	0.23	1.4	0.45	0.0025	0.0025	0.31	0.0075
37	CCC	MMM	Humoral	0.0025	0.0025	0.033	0.0075	0.04	0.0025	0.31	0.0075
38	CCC	MMM	Phagocytic	0.0025	0.0025	0.0075	0.0075	0.0025	0.0025	0.0075	0.0075
39	CCC	MMM	Phagocytic	0.070	0.25	0.36	0.84	0.026	0.0025	0.060	0.0075

* denotes results from samples taken after breakthrough infection. Negative S-specific and SNM-specific T cell results were imputed as 0.0025% and 0.0075% respectively. B, BNT162b2; C, CoronaVac; M (route), intramuscular; D, intradermal.

### Longitudinal analysis of antibody responses by vaccine brand and route, including Omicron BA.1 neutralization

**Figure 4:**
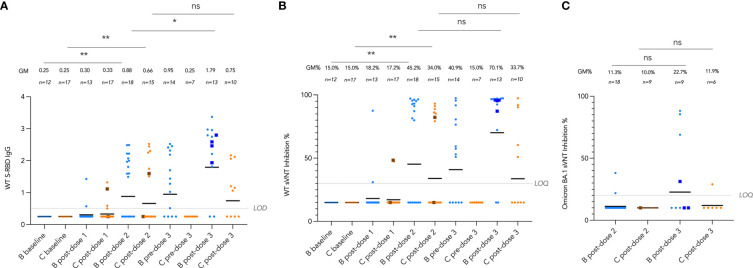
Longitudinal analysis of **(A)**, wild-type (WT) Spike-receptor binding domain (S-RBD) IgG, **(B)**, WT surrogate virus neutralization test (sVNT) results, and **(C)**, Omicron BA.1 sVNT by vaccine brand. Geometric means (GM) are shown with center lines and stated above each column. Limits of detection and quantification (LOD and LOQ) and cut-offs were drawn as grey lines. Data from participants receiving intradermal vaccination were shown as darkened squares beginning at their initial intradermal dose. Data from the same participant were analyzed longitudinally by paired t test after natural logarithmic transformation, and the P values are denoted by asterisks (*, P<0.05; **, P<0.01; ns, not significant).

**Figure 5:**
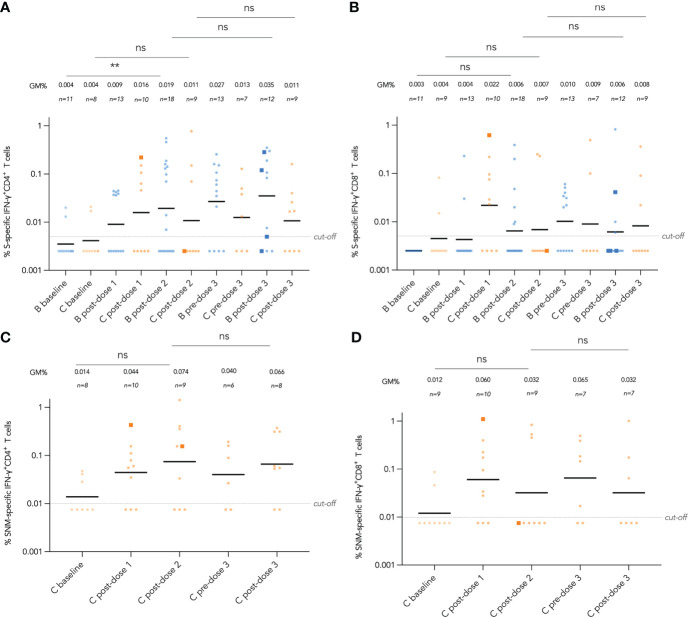
Longitudinal analysis of wild-type (WT) S and S, N and M protein peptide pool-specific interferon-γ (IFN-γ)^+^ CD4^+^ and CD8^+^ T cells by vaccine brand and route. S-specific IFN-γ^+^ CD4^+^ and CD8^+^ T cell responses are shown in **(A, B)** respectively for both BNT162b2 and CoronaVac recipients, while added SNM-specific IFN-γ^+^ CD4^+^ and CD8^+^ T cell responses are shown in **(C, D)** for CoronaVac recipients. Samples from the same patient were paired between baseline and post-dose 2 timepoints as well as post-dose 2 and post-dose 3 timepoints, and compared with paired t test after natural logarithmic transformation with p-values denoted (**, P<0.01; ns, not significant).

We tracked these antibody and T cell responses longitudinally from pre-vaccine baseline to post-dose 3 by vaccine brand in **Figures 4** and **5**. Very few (6 out of 30, 20%) participants seroconverted after a single dose of vaccine. Two doses of both vaccines significantly induced WT S-RBD IgG (both P<0.01) and WT sVNT (both P<0.01) (**Figure 4A, B**). Bloods were additionally drawn at pre-dose 3 for patients who received dose 3 at least 70 days (mean 150 days) after dose 2, and significant decline of sVNT levels was observed after BNT162b2 only (P=0.022). WT S-RBD IgG levels were also significantly increased by a third dose of BNT162b2 (P=0.037) and increasing trends of WT S-RBD for CoronaVac and WT sVNT for both vaccines were also observed post-dose 3 compared to post-dose 2. Neutralization responses in those who received an intradermal third dose appeared high (all four >85% inhibition), and all were higher than the respective GM by vaccine brand and dose.

Neutralization capacity of patient sera against Omicron BA.1 was evaluated by sVNT which found most patients who received 2 doses of vaccine (89% for BNT162b2 and 100% for CoronaVac) could not neutralize BA.1 (**Figure 4C**), indicating that BA.1 markedly evades neutralization in IEI patients. BA.1 sVNT levels were significantly increased by dose 3 of BNT162b2 (P=0.043) but not CoronaVac, although dose 3 CoronaVac did elicit BA.1 neutralization response in 1 out of 6 patients tested.

### Longitudinal analysis of T cell responses by vaccine brand and route

Longitudinal analysis of IFN-γ^+^ CD4^+^ and CD8^+^T cell responses against SARS-CoV-2 S peptide pool in BNT162b2 recipients and S, N and M peptide pools in CoronaVac recipients from baseline to post-dose 3 are shown in **Figure 5A-D**. Responses were compared longitudinally between baseline and post-dose 2 by vaccine brand, as well as post-dose 2 with post-dose 3 by paired t test after natural logarithmic transformation. We only found weak increases that were statistically insignificant, except between baseline and post-dose 2 in BNT162b2 recipients for S-specific IFN-γ^+^ CD4^+^ T cells (P=0.0064; **Figure 5A**). Analysis of IL-2^+^ CD4^+^ and CD8^+^ T cells also yielded similar findings (**Figure S4A-D**). Four patients received an intradermal dose 3 appeared to have a similar distribution with patients who received intramuscular vaccination.

### Breakthrough COVID-19 cases

Breakthrough COVID-19 reported by participants would be monitored for 3 years after vaccination. In early 2022, Hong Kong had its first major wave of COVID-19, dominated by the Omicron BA.2.2 variant. Seven participants, in the humoral (n=5), combined (n=1) and dysregulation (n=1) categories, had reported COVID-19 during that time after a partial or complete primary series (**Table 1**). All participants reported mild infections without need for hospitalization. Contingency analyses by Fisher exact test showed that proportion of infected participants did not differ by vaccine brand (B 15% *vs* C 21%, P=0.69), age group (adults 15% *vs* children 21%, P=0.69) or disease category (humoral 29% *vs* non-humoral 9%, P=0.21). Immunogenicity assessments were available for 3 of them pre- and post-infection (patients 13, 22, and 26), all of whom were seronegative for S-RBD IgG both immediately before and after infection (**Tables 2**, **3**).

## Discussion

Studying immunogenicity to COVID-19 vaccines enables us to understand protection conferred by vaccination on IEI patients. Three doses of BNT162b2 and CoronaVac were well-tolerated by our pediatric and adult IEI patients. Antibody responses to COVID-19 vaccines were found to be lowest in patients with humoral deficiencies, yet non-responders were also found in other IEIs not affecting adaptive immunity. Antibody responses were enhanced by a third dose of vaccine, especially cross-neutralization against SARS-CoV-2 variant Omicron. T cell responses were detected in many patients after vaccination, yet there is heterogeneity in responses. Patients who received intradermal vaccination appeared in general to have higher antibody levels but had similar T cell response, though sample sizes were small. Breakthrough infections with BA.2 were mild in vaccinated IEI patients.

Antibody responses were a primary outcome measured in many COVID-19 vaccine studies, including this study, as antibody responses have been shown to correlate with protection against symptomatic COVID-19 (31). Our finding of seroconversion failure in 45% patients after 2 doses of COVID-19 vaccines, which was reduced to 26% after a third dose, strongly support the need for a 3-dose primary series in patients with IEIs. Another study also found seroconversion failure was reduced from 39% to 24% after a third dose in a heterogeneous cohort of IEI patients with mostly humoral immunodeficiencies (32). Many of these IEI patients, especially those with humoral deficiencies, mounted a detectable T cell response to COVID-19 vaccines. While understudied and controversial in the virology and vaccinology fields (33), adaptive immunity against severe viral illnesses, except for enteroviruses, depends on T cells rather than B cells, as exemplified by recurrent and life-threatening viral infections in patients with combined immunodeficiencies but not in those with agammaglobulinemia (34). The presence of both common seroconversion and T cell response in triple-vaccinated IEI patients suggests 3 doses may be adequate for primary series in IEI patients in general. A fourth dose is likely required a few months after the primary series as a booster, dependent on circulation of SARS-CoV-2 in the community, degree of antibody waning, and potency of cellular memory.

Although numbers of participants in each IEI category was small, trends could be observed. For example, those with humoral immunodeficiencies developed the lowest S-RBD IgG and sVNT after 2 vaccine doses, seroconversion failure was also found in a patient with *STAT1* gain-of-function after 2 doses and another with SCN after 3 doses of vaccine as well. Both patients did not undergo HSCT, and neither was on immunosuppressive medications. While functional antibody deficiency is known to associate with *STAT1* gain-of-function (35), impairment of humoral immune response in phagocytic disorders is not well delineated. Within our study, 4 out of 4 patients with phagocytic disorders without a history of HSCT tested at pre-dose 2 (2 with SCN, 1 with X-linked chronic granulomatous disease, CGD, and 1 X-linked CGD carrier) did not seroconvert to a single dose of BNT162b2 (n=2) or a single dose of CoronaVac (n=2), which contrasts with the 100% seroconversion to a single dose of BNT162b2 in healthy adolescents (20). Of note, the carrier is mildly symptomatic with lupus-like facial rash. Our findings suggest a partially impaired B cell response to vaccines in patients with phagocytic disorders, which may be rescued by dose 2 or more additional doses. While conventional knowledge dictates vaccine response may only be impaired in patients with adaptive defects, seroconversion failure may be found in patients with different IEIs, and patients with any specific IEI should be recommended to complete the 3-dose primary series with booster vaccination, irrespective of disease category. Interestingly, we also observed seropositivity in several patients with humoral immunodeficiencies, including XLA, after 3 doses of vaccine, and in 2 XLA patients after just 2 doses. Whether this is genuine seroconversion due to residual B cell function, somatic reversion, or immunoglobulin replacement, remains to be further investigated.

CoronaVac is widely used globally, and this is likely to include patients with IEI patients as well, yet little data have been published on IEI patients. Studies in adult patients with secondary immunodeficiencies or immune dysregulation disorders showed reduced antibody responses to 2 doses of CoronaVac (36–38). S-RBD IgG and sVNT results in our study showed 3 doses of CoronaVac elicited antibody response in IEI patients. Eleven of 17 patients with humoral deficiencies in our study opted for CoronaVac. Whole-virion inactivated vaccines could elicit T cell response against other structural proteins such as N and M unelicited by S-only mRNA vaccines (20, 39), which correlate with protection against severe disease and infection (40, 41), and are not susceptible to mutations in Omicron (29). In our patients with humoral immunodeficiencies and no breakthrough COVID-19, 4 out of 5 patients in our study had a detectable SNM-specific IFN-γ^+^ CD4^+^ T cell response after just 2 doses of CoronaVac; the single patient who did not have a detectable IFN-γ^+^ CD4^+^ T cell response had an IL-2^+^ CD4^+^ T cell response. The effectiveness of CoronaVac in IEI patients was further supported by the 4 patients who received CoronaVac and experienced a mild breakthrough COVID-19. Our results support that CoronaVac is safe and effective in IEI patients.

We hypothesized intradermal vaccination may elicit better antibody and T cell responses in IEI patients. While there seemed to be no appreciable difference with T cell response of intradermal vaccination in IEI patients, we found a trend toward higher antibody responses in intradermal vaccinees. Additional studies involving healthy children or IEI patients may confirm our findings. We also examined the immunogenicity outcomes in patients receiving intradermal vaccination on a case-by-case basis as “n-of-1 trials”. Strikingly, one patient, who is a 15-year-old boy with post-HSCT X-SCID, did not seroconvert after receiving 3 doses of intradermal CoronaVac and contracting COVID-19 during the Omicron BA.2-dominant period in Hong Kong. Chart review revealed that the patient had a successful allogenic hematopoietic stem cell transplant with a 10/10 matched unrelated donor and conditioning regimen of fluradabine, melphalan, anti-thymocyte globulin and rituximab by the age of 1 year, resulting in 51% donor chimerism in the peripheral blood and him being generally healthy without immunoglobulin replacement. Last follow-up showed normal T and B cell counts (1829 cells/ul and 406 cells/ul) and normal IgG level (1289 mg/dl). The patient also previously seroconverted to intramuscular vaccines with seropositivity for measles, mumps, rubella, and tetanus. He has been on penicillin prophylaxis after splenectomy at age of 1 year as disseminated BCGosis involved his spleen. Interestingly, 2 years prior to COVID-19 vaccination, the patient developed cryotherapy-resistant verruca vulgaris on his right hand, a known complication after HSCT in X-SCID patients. That led us to hypothesize that seroconversion failure to intradermal vaccine is also due to mutated keratinocytes in the skin, with impaired chemotactic functions, not corrected by HSCT (42, 43). This finding suggests that while intradermal vaccination may enhance seroconversion in most immunocompromised vaccinees, X-SCID patients who underwent HSCT may not benefit from intradermal vaccination.

Our study had several strengths and limitations. In addition to antibodies, we also studied T cell responses, which protect against disease progression. We were able to track both antibody and T cell responses sequentially in vaccinees from pre-vaccine to post-dose 3. The utility of longitudinal antibody testing in predicting infections deserves to be studied in larger multi-cohort studies. We did not recommend an interval between immunoglobulin replacement administration and vaccination or blood sampling, as we did not believe immunoglobulin replacement would affect the immunogenicity of either vaccine, and to avoid disturbance to patients’ treatment. Immunoglobulin replacement preparations were not tested to rule out the effect of passive immunization. Our sample size was limited due to rarity of IEIs, yet studies from other centers could corroborate our findings. Future studies or meta-analyses of similar studies could also determine the effect of immunomodulators on immunogenicity. We could not assess clinical effectiveness.

In conclusion, our findings support the need for 3 doses of mRNA or inactivated COVID-19 vaccines for IEI patients and this should be regarded as the primary series of vaccination. Future studies should focus on the longevity of immune response and effect of a fourth dose and hybrid immunity in these patients.

## Data availability statement

The original contributions presented in the study are included in the article/**Supplementary Material**. Further inquiries can be directed to the corresponding authors.

## Ethics statement

This study was reviewed and approved by University of Hong Kong. Written informed consent to participate in this study was provided by the participants’ legal guardian/next of kin. Written informed consent was obtained from the individual(s), and minor(s)’ legal guardian/next of kin, for the publication of any potentially identifiable images or data included in this article.

## Authors contribution

YL conceptualized the study. YL, MP, WT, DL, and JD designed the study. YL led the acquisition of funding. YL, WT and MP supervised the project. SMC, DL, XM, SMSC, IT and JL led the study administrative procedures. HW provided software support. SMC and HW contributed to recruitment of participants. YL and JD provided study-related clinical assessments and follow-up. DL, SMC, CC, JL, JD and YL collected safety data. SMSC, SC, LT and MP developed and performed S-RBD IgG, and sVNT. XM, YZ, MW, WZ, YC, HW, AL, WL and WT developed and performed the T cell assays. DL, TSL, and JL curated and analyzed the data. DL and JL visualized the data. DL, XM, SMSC, JD, JL and SMC validated the data. JD, GC, K-NC, EA, JK, PC, PL, MH, TLL and YL provided clinical care. DL wrote the first draft supervised by YL, with input from JD, XM and SMSC. All authors contributed to the article and approved the submitted version.

## Funding

This study was supported by the research grants COVID19F02, COVID19F10, and COVID19F12 from the Hong Kong SAR Government, which was not involved in the study design, performance, interpretation, or publication of this project.

## Acknowledgments

We thank the staff at Community Vaccination Centers at Ap Lei Chau Sports Centre, Gleneagles Hospital Hong Kong, and Sun Yat-Sen Memorial Park Sports Centre. The investigators are grateful to all clinical research team members and laboratory staff of Department of Pediatrics and Adolescent Medicine of the University of Hong Kong, for their research support. We are most thankful to the study participants, as well as clinicians and laboratory staff who have provided clinical care and testing to the patients.

## Conflict of interest

The authors declare that the research was conducted in the absence of any commercial or financial relationships that could be construed as a potential conflict of interest.

## Publisher’s note

All claims expressed in this article are solely those of the authors and do not necessarily represent those of their affiliated organizations, or those of the publisher, the editors and the reviewers. Any product that may be evaluated in this article, or claim that may be made by its manufacturer, is not guaranteed or endorsed by the publisher.
