# Supplementary material for: Safety and immunogenicity of 3 doses of BNT162b2 and CoronaVac in children and adults with inborn errors of immunity
Source: Front Immunol. 2022 Sep 20;13:982155. doi: 10.3389/fimmu.2022.982155 (PMC9530261; doi:10.3389/fimmu.2022.982155)
Supplement: Supplementary file 1 [file DataSheet_1.docx]

**SUPPLEMENTAL METHODS**

*S-RBD IgG and surrogate virus neutralization test (sVNT)*

The SARS-CoV-2 S receptor-binding domain (S-RBD) IgG enzyme-linked immunosorbent assay (ELISA) were carried out as previously described and validated.(1) sVNT was conducted according to the manufacturer’s instructions (GenScript Inc, Piscataway, USA) and as described in our previous publications.(2) All sera were heat-inactivated at 56° C for 30 minutes before testing.

In brief, S-RBD IgG ELISA plates were coated overnight with 100 ng/well of purified recombinant S-RBD in PBS buffer, followed by addition of 100 μL Chonblock Blocking/Sample Dilution (CBSD) ELISA buffer (Chondrex Inc, Redmond, USA). This was incubated at room temperature (RT) for 2 hours. Serum was tested at a dilution of 1:100 in CBSD ELISA buffer, then added to the wells for 2 hours at 37°C. After washing with PBS containing 0.1% Tween 20, horseradish peroxidase (HRP)-conjugated goat anti-human IgG (1:5,000) (GE Healthcare, Chicago, USA) was added for 1 hour at 37°C, followed by washing five times with PBS containing 0.1% Tween 20. HRP substrate (Ncm TMB One, New Cell & Molecular Biotech Co. Ltd, China) of 100 μL was added for 15 minutes, and the reaction was stopped by 50 μL of 2 M H_2_SO4. The OD was analysed in a Sunrise absorbance microplate reader (Tecan, Männedorf, Switzerland) at 450 nm wavelength. The background OD in PBS-coated control wells with the participant’s serum was subtracted from each OD reading. Values at or above an OD450 of 0.5 were considered positive and values below were imputed as 0.25.

The sVNT was performed using 10 μL of each serum, positive and negative controls, which were diluted at 1:10 and mixed with an equal volume HRP conjugated to the WT or BA.1 SARS-CoV-2 S-RBD (6 ng). The mixture was incubated for 30 minutes at 37°C, then 100 μL of each sample was added to microtitre plate wells coated with angiotensin-converting enzyme-2 (ACE-2) receptor. This plate was sealed for 15 minutes at 37°C and then washed with wash-solution, tapped dry, and 100 μL of 3,3',5,5'-tetramethylbenzidine (TMB) was added and incubated in the dark at RT for 15 minutes. This reaction was stopped with 50 μL of Stop Solution and the absorbance at 450 nm was detected by a microplate reader. The % inhibition of each serum was calculated as (1 - sample OD value/negative control OD value) x100%. Inhibition (%) of at least 30%, the limit of quantification (LOQ), was regarded as positive, and values below 30% were imputed as 15%.

*T cell responses*

T cell responses were tested as previously published.(3, 4) Peripheral blood mononuclear cells (PBMCs) were isolated from whole blood by density gradient separation then frozen in liquid nitrogen until use. Thawed PBMCs were rested for 2 hours in 10% human AB serum supplemented RPMI medium. Next, the cells were stimulated with sterile ddH2O or 1 µg/mL overlapping peptide pools representing the WT SARS-CoV-2 S, N and M proteins (Miltenyi Biotec, Bergisch Gladbach, Germany) for 16 hours in the presence of 1 µg/mL anti-CD28 and anti-CD49d costimulatory antibodies (clones CD28.2 and 9F10, Biolegend, San Diego, USA). After 2 hours of stimulation, 10 µg/mL brefeldin A (Sigma, Kawasaki, Japan) was added. The cells were then washed and subjected to immunostaining using a fixable viability dye (eBioscience, Santa Clara, USA, 1:60) and antibodies against CD3 (HIT3a, 1:60), CD4 (OKT4, 1:60), CD8 (HIT8a, 1:60), IFN-γ (B27, 1:15) and IL-2 (MQ1-17H12, 1:15) antibodies (Biolegend, San Diego, USA). Data acquisition was carried out using flow cytometry (LSR II; BD Biosciences, Franklin Lakes, USA) and analyzed by Flowjo v10 software (BD, Ashland, USA). The antigen-specific IFN-γ^+^ or IL-2^+^ T cells were calculated by subtracting the background (sterile ddH2O) data, and presented as the percentage of CD4^+^ or CD8^+^ T cells. T cell response against a single peptide pool was considered positive when the frequency of cytokine-expressing cells was higher than or equal to 0.005% and the stimulation index was higher than 2; negative values were imputed as 0.0025%. Total T cell responses against S, N and M peptide pools were also added together, with a cut-off of 0.01%.

**
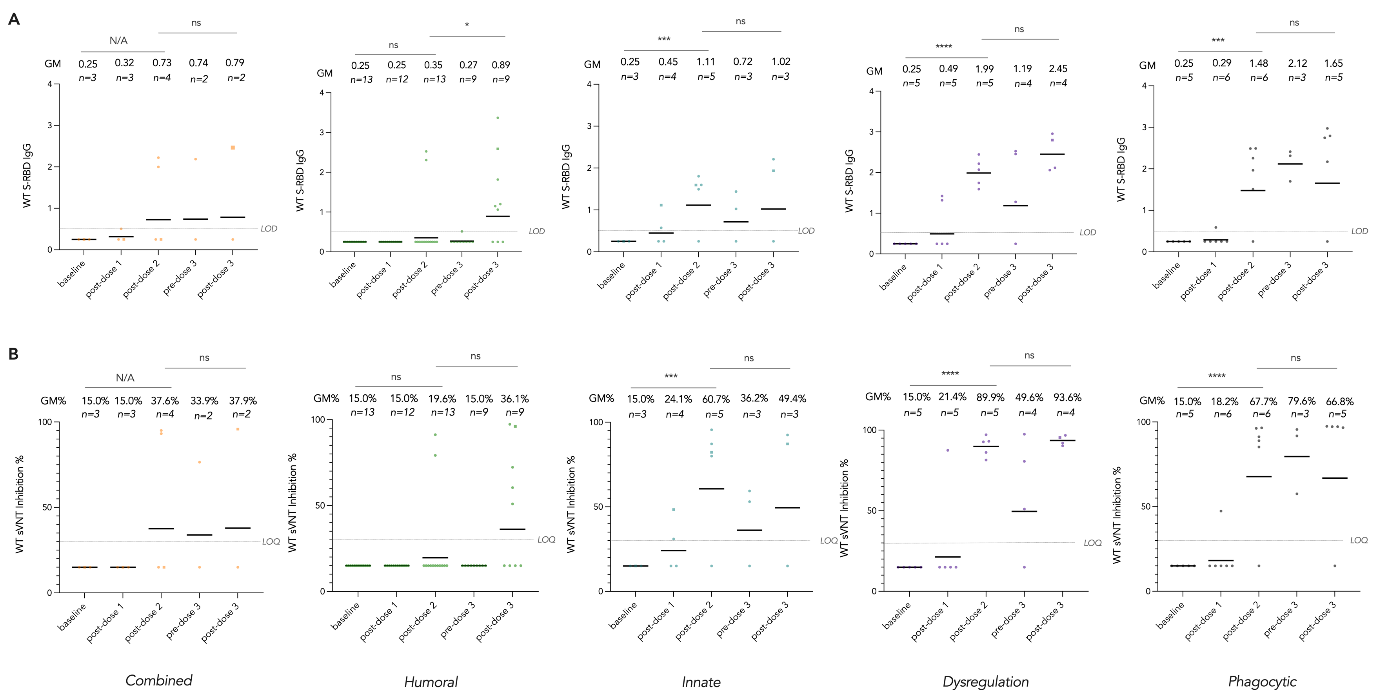
**

**FIG. S1. Longitudinal analysis of wild-type (WT) Spike-receptor binding domain (S-RBD) IgG and surrogate virus neutralization test (sVNT) results after COVID-19 vaccination by disease category.** A shows S-RBD IgG results while B shows sVNT results. Geometric means (GM) are shown with center lines and stated above each column. Limit of detection (LOD) and of quantification (LOQ) were drawn as grey lines. Number of analyzed available samples (n) are also stated above each column. Data from participants receiving intradermal vaccination were shown as darkened squares beginning at their initial intradermal dose. Samples from the same patient were paired between baseline and post-dose 2 timepoints as well as post-dose 2 and post-dose 3 timepoints, and compared with paired t test after natural logarithmic transformation with p-values denoted (*, P<0.05; **, P<0.01; ***, P<0.001; ****, P<0.0001; ns, not significant; N/A, not applicable).

**
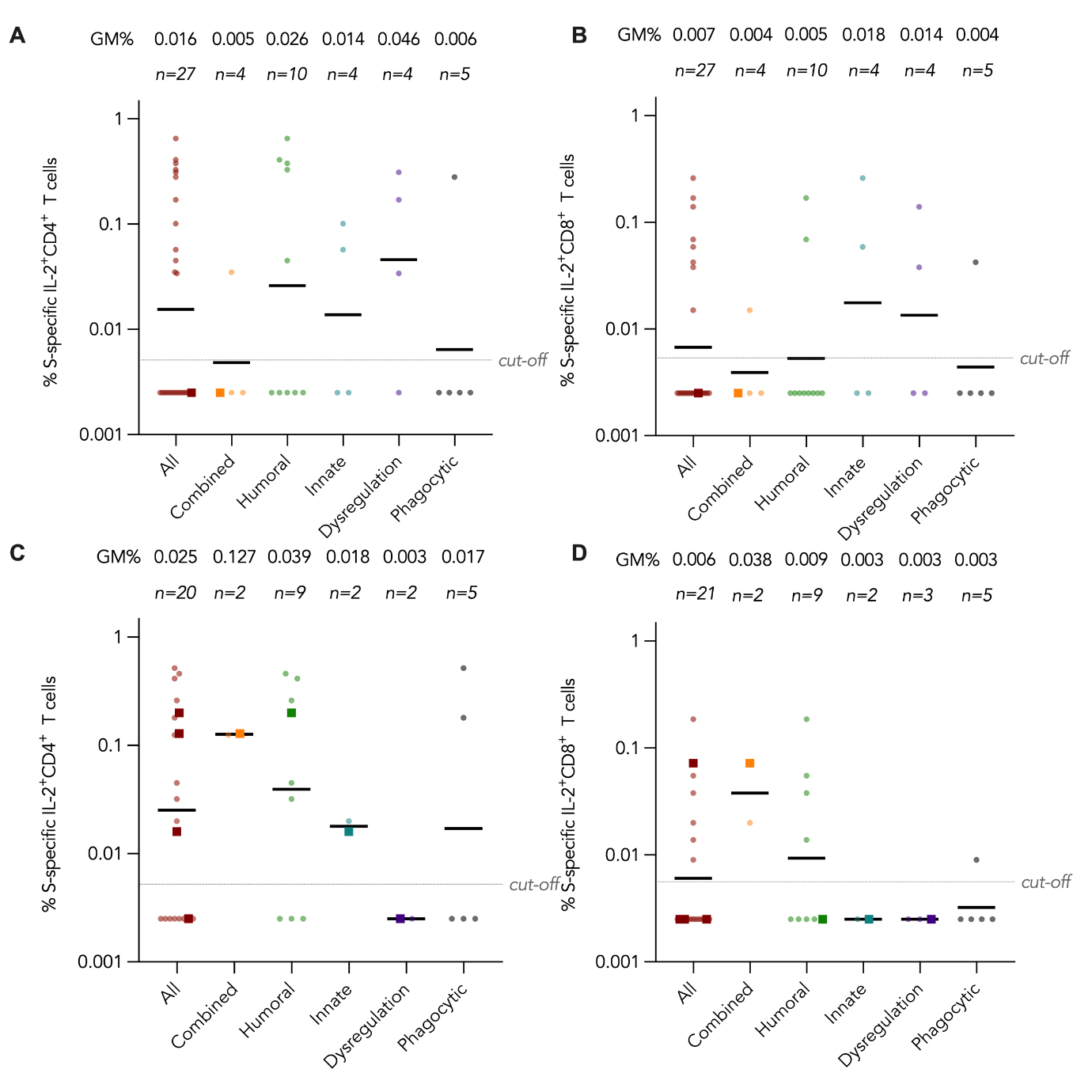
 FIG. S2. Wild-type (WT) S peptide pool-specific interleukin-2 (IL-2)^+^ CD4^+^ and CD8^+^ T cells after COVID-19 vaccination by disease category.** A and B show S-specific IL-2^+^ CD4^+^ and CD8^+^ results respectively in five disease categories (combined, humoral, innate, dysregulation and phagocytic) after 2 doses, while C and D show S-specific IL-2^+^ CD4^+^ and CD8^+^ results after 3 doses. Geometric means (GM) are shown with centre lines and stated above each column. Cut-offs were drawn as grey lines. Number of analyzed available samples (n) are also stated above each column. A and B included 1 patient (in combined) who received first 2 doses intradermally and their datapoints were shown as darkened squares. C and D also included 4 patients (1 each in combined, humoral, innate and dysregulation) who received their third dose intradermally and their datapoints were also shown as darkened squares.


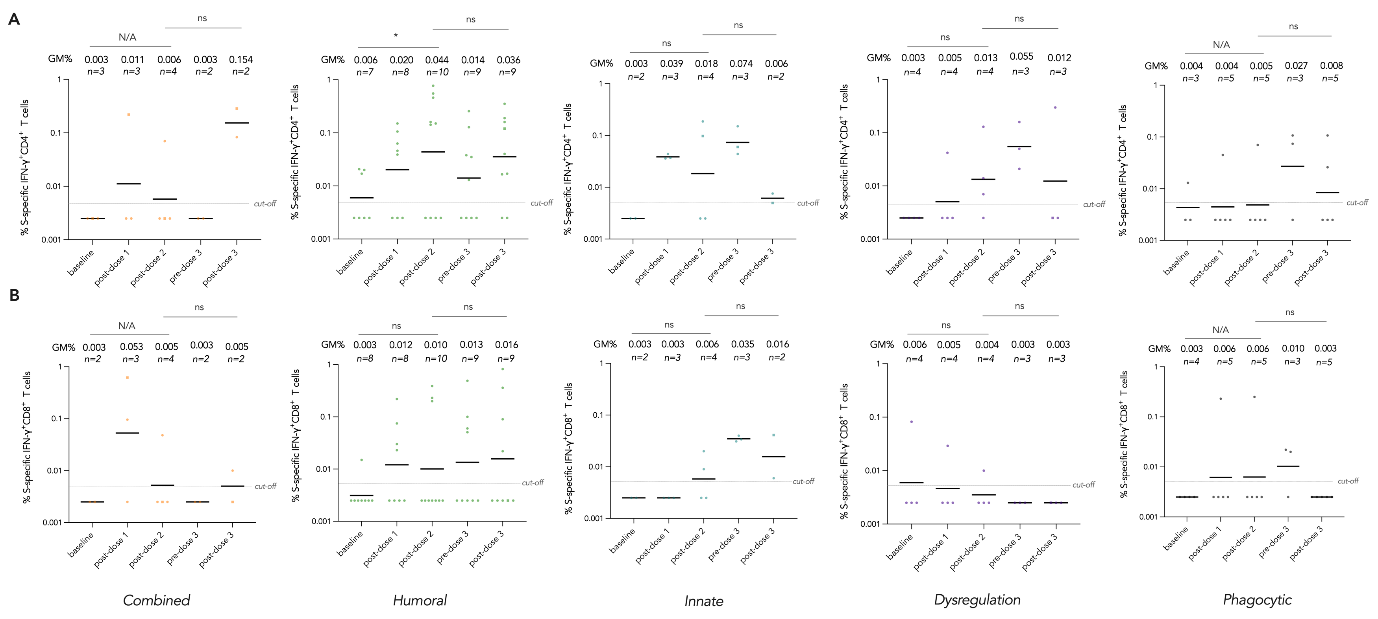


**FIG. S3. Longitudinal analysis of S peptide pool-specific interferon-γ (IFN-γ)^+^ CD4^+^ and CD8^+^ T cells after COVID-19 vaccination by disease category.** A shows IFN-γ^+^ CD4^+^ T cells results while B shows IFN-γ^+^ CD8^+^ T cells results. Geometric means (GM) are shown with center lines and stated above each column. Cut-offs were drawn as grey lines. Number of analyzed available samples (n) are also stated above each column. Data from participants receiving intradermal vaccination were shown as darkened squares beginning at their initial intradermal dose. Samples from the same patient were paired between baseline and post-dose 2 timepoints as well as post-dose 2 and post-dose 3 timepoints, and compared with paired t test after natural logarithmic transformation with p-values denoted (*, P<0.05; ns, not significant; N/A, not applicable).


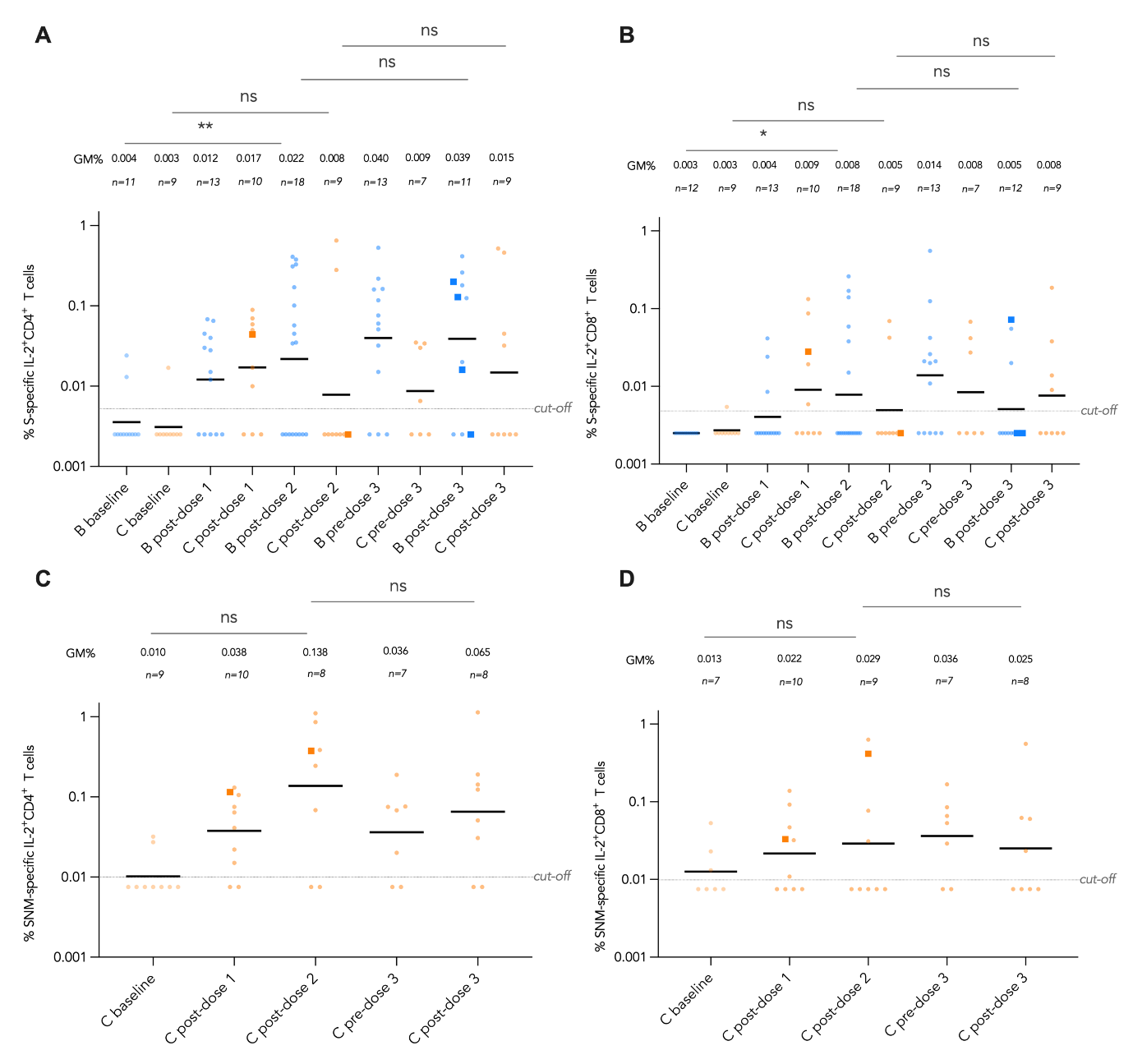


**FIG. S4. Longitudinal analysis of wild-type (WT) S and S, N and M protein peptide pool-specific IL-2 (IL-2)^+^ CD4^+^ and CD8^+^ T cells by vaccine brand and route.** S-specific IL-2^+^ CD4^+^ and CD8^+^ T cell responses are shown in A and B respectively for both BNT162b2 and CoronaVac recipients, while added SNM-specific IL-2^+^ CD4^+^ and CD8^+^ T cell responses are shown in C and D for CoronaVac recipients. Samples from the same patient were paired between baseline and post-dose 2 timepoints as well as post-dose 2 and post-dose 3 timepoints, and compared with paired t test after natural logarithmic transformation with p-values denoted (*, P<0.05; **, P<0.01; ns, not significant).

**SUPPLEMENTAL REFERENCES**

1. Perera RA, Mok CK, Tsang OT, Lv H, Ko RL, Wu NC, et al. Serological assays for severe acute respiratory syndrome coronavirus 2 (SARS-CoV-2), March 2020. Euro Surveill. 2020;25(16).

2. Perera R, Ko R, Tsang OTY, Hui DSC, Kwan MYM, Brackman CJ, et al. Evaluation of a SARS-CoV-2 Surrogate Virus Neutralization Test for Detection of Antibody in Human, Canine, Cat, and Hamster Sera. J Clin Microbiol. 2021;59(2).

3. Rosa Duque J, Wang X, Leung D, Cheng S, Cohen C, Mu X, et al. Immunogenicity and reactogenicity of SARS-CoV-2 mRNA and inactivated vaccines in healthy adolescents (Accepted). Nat Commun. 2022.

4. Leung D, Cohen CA, Mu X, Rosa Duque J, Cheng SM, Wang X, et al. Immunogenicity Against Wild-Type and Omicron SARS-CoV-2 After a Third Dose of Inactivated COVID-19 Vaccine in Healthy Adolescents. SSRN: Cell Press Sneak Peek. 2022.
